# Radiotherapy in Malignant Disease in the Neighborhood of the Eye

**Published:** 1906-11

**Authors:** Bernard Wolff

**Affiliations:** Atlanta, Ga.


### RADIOTHERAPY IN MALIGNANT DISEASE IN THE NEIGHBORHOOD OF THE EYE.*

BY BERNARD WOLFF, M.D.,

ATLANTA, GA.

I wish to present this short paper as a plea for the more general employment of the X-rays in malignant affections of the structures in the neighborhood of the eye, especially epithelioma of the lids.

The sphere of usefulness of this agent has been fairly well defined. After many extravagant claims which appeared in the earlier history of the method had been disqualified by fact and experience, it has been pretty well established that in certain conditions on the frontier of malignancy, the X-rays are the most satisfactory as well as reliable means of relief.

When one considers the delicacy of the eye, the importance of its unimpaired functioning and the necessity of preserving the integrity of the lids so far as practicable, it will readily be seen that surgery should be applied to this locality with great circumspection. In excision there is left a breach which filling up with cicatricial tissue contracts and disturbs the apposition of the palpebral margins with the attendant ills. The results of plastic operations when recent are often sightly enough, but as time passes and the contractile quality of scar tissue asserts itself, the remote outcome from a cosmetic standpoint leaves much to be desired.

The objection to the use of caustics is even greater than to excision. The action of the caustic is with difficulty circumscribed and the cicatrix resulting may produce ineradicable deformity. There is no special reason why, with very small growths, erosion with the curette and cauterization with acid nitrate of mercury should not be practiced, except that the infliction of even a small amount of pain and discomfort is by many people greatly dreaded.

*Read before the Fifth District Medical Society, October 16, 1906.

Epithelioma of the lid and its vicinage is prone to occur in one of two forms. The most frequent perhaps is that seen in old people. It begins as a scaly spot or patch, which after years of duration, becomes crusted, then active, ulcerates and finally takes on the characteristic features of malignant disease. This type is usually seen on the free surface of the lower lid near one or the other angle, on the temple near the outer angle or on the nose close to the inner canthus. It is slow in development and course.

Another form is seen principally in middle-aged people and manifests itself as a waxy papule or tubercle on the ciliary margin of the lid. It may project upward among the lashes or lie flattened on the conjunctival border and encroach upon the conjunctiva. It is not to be mistaken for the wart that is frequently seen upon the lid. This form of epithelioma runs a more rapid course than the foregoing, grows more rapidly and ulcerates earlier. Both have small beginnings, but are so situated that a cutting operation will inevitably produce scar tissue and more or less disfigurement.

As one's personal experience is always much more instructive and convincing to him than that of another, I am frank to say that from the cases which I have observed I would never resort to a cutting operation in malignant disease of the lids as the first move in securing relief, but would hold it in retirement to be brought forward only in the event of the failure of the X-rays to secure the desired result. The only serious objection that with any force may be lodged against the employment of the X-rays is the leisurely character of response to the treatment. To cause a disappearance of the growth many exposures are often required. One can not state even approximately how many an individual case may require. The range may be from ten to one hundred, or even more. The majority of cases treated by me have recovered within thirty days from the beginning of the treatment, having taken daily or tri-weekly sittings. Epithelioma is especially frequent in those who have suffered from seborrhea of long standing, and individuals who possess an oily loose skin are particularly responsive to radiotherapy. In carrying out the treatment some protection should be afforded to the eye, although there is not much risk of injuring it, provided the lid can be closed over it and the tube be not too heavily energized.

Any material which is impermeable to the X-rays will serve. Lead foil is serviceable as well as many of the shield devices. The most convenient form of shield with which I am acquainted is that made by Wagner, of Chicago. It is of flexible rubber, fitting over the tube and is provided with metal diaphragms with openings of different sizes. This arrangement protects both patient and operator. The same kind of shield may be improvised by using electrician's adhesive tape and covering the tube except for a foramen for the emergence of the rays.

The tube may be approached so near the eye that the lids infringe upon the metal rim and the rays are delivered accurately upon a small surface. If the growth is concealed when the lid is closed, the latter may be drawn down by a strip of adhesive plaster, attaching the other end to the cheek. This will hold it open long enough for a five or ten minutes sitting. The treatment if possible should be given daily. After a varying length of time the skin becomes dusky red, and somewhat scaly. This constitutes the so-called X-ray dermatitis, which, as a rule, passes off in a week or ten days. The growth may disappear with it or shortly after. Occasionally the reaction is somewhat more intense, and there may be some crusting, but this is a matter of no moment, as it soon passes off and does not damage the skin to any appreciable extent.

I have never had any serious results following the use of the X-rays and, in fact, none that occasioned the patient or myself any apprehension.

The use of the X-rays may be reasonably extended to malignant disease of the orbit, and sarcoma, gumma and perhaps some non-malignant pathological states such as trachoma, corneal ulcer, chronic comjunctivitis and the like. To sum up: The X-rays may be depended upon with fair constancy to relieve superficial epithelioma of the lid and its vicinity; it is painless, produces no scar, and in the hands of a cautious or experienced person, is free from risk.

